# Canine salivary gland carcinoma treated with stereotactic body radiation therapy: a retrospective case series

**DOI:** 10.3389/fvets.2023.1202265

**Published:** 2023-06-27

**Authors:** Patricia Gualtieri, Tiffany Martin, Del Leary, Susan E. Lana, Susan M. LaRue, Mary-Keara Boss

**Affiliations:** ^1^Department of Environmental and Radiological Health Sciences, College of Veterinary Medicine and Biomedical Sciences, Colorado State University, Fort Collins, CO, United States; ^2^Department of Clinical Sciences, College of Veterinary Medicine and Biomedical Sciences, Colorado State University, Fort Collins, CO, United States

**Keywords:** dog, neoplasia, radiotherapy, metastasis, lymph node

## Abstract

**Objective:**

The aim of this study was to describe the therapeutic outcomes of dogs with locally advanced salivary gland carcinomas (SGC) following stereotactic body radiation therapy (SBRT).

**Methods:**

A single institution retrospective study was conducted of client-owned dogs with macroscopic SGC treated with SBRT. Patient signalment, clinical characteristics, and treatment parameters were recorded. Clinical benefit was determined based on follow-up physical examination and medical history. Progression-free interval (PFI), median survival time (MST), and disease-specific survival (DSS) were calculated using Kaplan–Meier analysis. Acute and late toxicity were recorded according to Veterinary Radiation Therapy Oncology Group (VRTOG) criteria.

**Results:**

Six patients were included in the study. Tumor origins were mandibular (*n* = 3), parotid (*n* = 2), and zygomatic (*n* = 1) salivary glands. The SBRT prescription was 10 Gy × 3 daily or every other day. All patients (100%) experienced clinical benefit from treatment at a median time of 34 days (range 28–214). No local or regional nodal failure was reported following SBRT. Progressive pulmonary metastatic disease was documented in three dogs (50%). The median PFI was 260 days (range 43–1,014) and the MST was 397 days (range 185–1,014). Median DSS was 636 days (range 185–1,014). Four dogs (66.6%) died of confirmed or suspected metastatic SGC. The reported acute side effects included grade 2 mucositis (*n* = 1) and vision loss (*n* = 1). No late side effects were recorded.

**Conclusion:**

This study suggests that SBRT may provide durable local control for invasive SGC in dogs. Further investigation in a larger cohort of patients is warranted. The incidence of reported acute and late toxicity was low.

## 1. Introduction

Salivary gland neoplasia is considered a rare disease in dogs; however, it accounts for reported ranges of 20.1–30% of submitted pathologic salivary gland tissue (1–3). The majority (88.8%) of neoplasms described are of epithelial origin (3). There is sparse information regarding clinical characteristics, treatment management, and outcome for dogs with salivary gland tumors (4).

In human medicine, surgery is the mainstay treatment, with adjuvant postoperative radiation therapy (RT) recommended in cases of high-grade histology, cervical lymph node status, advanced stage, bony or perineural invasion, and/or inadequate excision to maximize local control and overall survival (5, 6). Surgical excision and adjuvant RT obtain high locoregional control rates for human SGC patients (5, 6). In veterinary medicine, a local recurrence rate of 42% of cases has been described in canine SGC treated with surgical excision alone, with no correlation identified between capsular invasion or margin status and local recurrence, and an overall progression free interval (PFI) of 191 days (7). In that retrospective study, lymph node metastasis was present in 28.9% at the time of surgery and these dogs had a shorter PFI at 98 days and a DSS time of 248 days (overall MST was 498 days; DSS without lymph node metastasis was 1,886 days).

For human SGC patients, RT alone can be considered as a reasonable alternative definitive-intent treatment option in patients with inoperable disease, patients at high risk of complications because of comorbidities, and patients who have refused surgery (6, 8). RT can also represent a palliative treatment option in the case of distant metastases (6, 8–10). Specifically, SBRT has been reported to have a role in human SGC patients with local invasion, gross disease with one or more adverse features, and yields good local control rates and acceptable toxicity when used as boost treatment to the gross tumor volume in combination with conventionally fractionated IMRT and concurrent platin-based chemotherapy (10). Additionally, SBRT has been investigated for its potential role in the management of oligometastatic SGC patients to control limited burden of disease, specifically in cases of ≤3 metastatic lesions most commonly affecting lung, bone, and brain metastasis (11).

In veterinary medicine, RT has been reported in both dogs and cats as an adjunctive treatment to surgery for SGC to achieve local tumor control, which may or may not be administered in combination with chemotherapy (12, 13). Local control rates of 100% have been described in a case series of three dogs with incomplete surgical excision of SGC that were treated with adjuvant fractionated orthovoltage RT in with a total dose of 45 Gy delivered in 10 fractions and a follow up time of 9, 25, and 40 months (12).

At the time of preparing this manuscript, there was no published literature regarding local control of canine SGC treated with external beam RT alone in the macroscopic disease setting. The aim of this study was to describe the feasibility, safety profile, and outcome following SBRT for locally advanced SGC in dogs.

## 2. Materials and methods

### 2.1. Case selection and medical record review

A single institution retrospective study was performed. The inclusion criteria for the study were dogs with SGC based on cytologic or histopathologic diagnosis and identified to be salivary in origin according to computer tomography (CT) imaging. All patients were treated with external beam SBRT between August 2010 and December 2021. Specifically, the patients had to be treated with three to five fractions of SBRT for a total prescribed dose of 30 Gy. Exclusion criteria included carcinoma of locations other than salivary gland and microscopic disease setting. Full staging was not required for inclusion in this study.

Medical records were reviewed to collect patient demographics including: age, sex, breed, weight, clinical signs at time of diagnosis, date of diagnosis, presence/absence of peripheral lymphadenopathy based on physical exam by attending clinician, concurrent comorbidities, and any tumor types other than salivary gland carcinoma present at the time of treatment. Medical and surgical management of the salivary gland carcinoma prior to presentation for radiation therapy were also recorded. Gross tumor specific characteristics included: specific tumor location and salivary gland affected, tumor size based on physical examination and caliper measurement of longest tumor diameter, and cytologic and/or histopathologic characteristics.

### 2.2. Staging and diagnostic testing

Stage of the salivary tumor was determined according to the Tumor, node, metastasis (TNM) staging system (14). Data recorded from staging diagnostics prior to radiation therapy included: complete blood count and chemistry within 1 month of the first anesthesia event, cross sectional imaging of the primary tumor via CT with radiation planning, thoracic imaging with 3-view thoracic radiographs, and/or thoracic CT. Thoracic CT images were obtained during expiratory breath-hold after temporary hyperventilation. Locoregional lymph node cytology and histopathology findings were recorded, when available. Sampling of locoregional lymph nodes was dependent on attending clinician’s discretion. Abdominal imaging and additional imaging modalities performed (e.g., magnetic resonance imaging, echocardiography) were also recorded, when available.

CT scans were performed using either a Philips Gemini TF Big Bore 16-slice scanner (Philips Medical Systems, Nederland, B.V.) or a Siemens Somaton Force 192-slice scanner (Siemens Medical Solutions, Pennsylvania). All dogs were positioned in sternal recumbency, with forelimbs positioned caudally. The head and cervical region were immobilized using a carbon fiber stand, a fixed personalized dental mold (Exaflex Putty, GC America Inc., Alsip, IL), a thermoplastic bead facial mask, and a vacuum bead style moldable cushion for ventral neck support, as previously described (15). A non-contrast helical scan was performed of the skull through to thoracic inlet, with a postcontrast scan performed after intravenous injection of Omnipaque 350 contrast media (GE Healthcare, Princeton, New Jersey; 2.2 mL/kg). Images were reconstructed in 2.0 mm contiguous intervals with 512 matrix and smooth algorithm.

Tumor stage was determined based on retrospective evaluation of CT scans interpreted by American College of Veterinary Radiology-certified radiologists. Primary tumor characteristics recorded included largest tumor diameter and presence of invasion in surrounding tissues. Lymph node size (normal vs. enlarged), contrast-enhancing pattern (homogenous vs. heterogenous), and effacement by tumor, as interpreted by the board-certified radiologist, were recorded. Soft tissue pulmonary nodules identified on thoracic radiographs or CT scan were assumed distant metastasis and were not confirmed by cytology or histology.

### 2.3. Radiation planning, treatment delivery, and plan parameters

Both the 2 mm pre-contrast and post-contrast CT scans were imported and utilized for inverse treatment planning with the Varian Eclipse treatment planning system (Varian Medical Systems, Inc. Palo Alto, California). Gross tumor volume (GTV) and organs at risk (OARs) were identified and contoured. A planning target volume (PTV) incorporated a 2–5 mm isotropic expansion from the GTV to account for daily set-up error for positioning, with the expansion being determined by the attending radiation oncologist’s discretion. Locoregional lymph node inclusion was based on clinical and imaging-based concern at the discretion of the attending radiation oncologist. Gross tumor volume of the lymph node(s) was included in the primary tumor GTV when effaced or in close proximity of the primary GTV, or contoured separately (GTVn), with a nodal planned target volume (PTVn) of 2–5 mm isotropic expansion from the GTVn or 4–9 mm asymmetric expansion for mandibular and retropharyngeal lymph nodes as previously described (16). The OARs included skin, trachea, esophagus, spinal cord, eyes, lenses, brain, and optic chiasm. The normal tissue constraints for the OAR were adopted and modified for canine patients from a previous report of human OAR constraints (17).

SBRT treatment plans were created with coplanar or non-coplanar 6 MV modulated static radiation beams or volumetric modulated arc therapy (VMAT). Radiation beams were modulated using the sliding-window technique. Radiation plans, at the time of development, were assessed based on the intent to deliver 100% of the radiation prescription to 99% of the GTV and 95% of the PTV. All dose predictions were made with Varian’s AAA dose calculation (version 15.6.) and optimization was done using Varian’s Photon Optimizer (version 15.6.06). PTV-less structures were created when indicated to remove normal OAR structures from the PTV and utilized in plan optimization and evaluation. Quality assurance was performed by gamma analysis using a Varian portal dosimetry system on individual fields and VMAT arcs. A passing QA score was required before treatment, with a minimum of 95% gamma for a 3 mm distance to agreement and a 3% absolute dose difference.

All patients were treated under general anesthesia. Protocols for anesthesia were variable, and often included a benzodiazepine drug such as midazolam (Midazolam – Injection USP 50 mg/10 mL, Hikma Pharmaceuticals Inc., New Jersey, United States) and propofol (Propoflo^™^ 100 mg/20 mLs, Zoetis, New Jersey, United States) for induction, and maintained on isoflurane gas once intubated. Daily patient positioning was verified by using on-board kV cone-beam CT. A 4 degrees-of-freedom couch was used to correct the position errors. Once positioning was confirmed, treatment was delivered using a Varian Trilogy linear accelerator (Varian Medical Systems, Inc. Palo Alto, California, United States).

Radiation plans were later assessed in a retrospective manner with the following data collected: GTV, PTV with corresponding less structures when available, volumes and doses to OARs, dose to 99 and 98% of GTV, dose to 95% of PTV, median, near minimum and near maximum tumor dose, conformity index (CI), heterogeneity index (HI), and gradient measure (GM), as previously recommended (18–21).

### 2.4. Response, toxicity evaluation, and clinical follow-up

Follow-up data recorded included clinical signs after treatment, acute and late normal tissue toxicities, response to therapy based on imaging and/or physical exam, and dates and results of follow up thoracic imaging, as well as date and cause of death. Post-SBRT chemotherapy and anti-inflammatory therapy was recorded. If not available, additional follow up information was obtained via electronic communication or phone interview with referring veterinarians and clients. Medical management post-RT was at the discretion of the medical team managing the individual case at the time of each visit. The intent of the authors was to assess treatment response according to RECIST criteria (22), utilizing physical exam and caliper and imaging measurements, when available. Clinical benefit was defined as a stable or reduced tumor size, a lack of mass-effect on follow up physical examination by a veterinarian, and/or improvement of clinical signs associated with the mass reported by the owner in the medical history. The time-to-improvement was defined as time from the start of SBRT treatment to the time of clinical benefit onset.

Normal tissue toxicity grading was recorded according to the Veterinary Radiation Therapy Oncology Group (VRTOG) morbidity scoring scheme for acute and late radiation effects (23), with acute effects being defined as signs occurring within the RT field within 90 days after treatment, and late effects being defined as after 90 days after therapy. Toxicities were determined based on medical record review and graded based on available information.

### 2.5. Statistical analysis

Patient outcomes were reported as progression-free intervals (PFI), median overall survival time (MST), and disease-specific survival (DSS). The PFI was calculated from the first day of treatment to the day of local or distant progression. MST was calculated from the first day of treatment to the time of death of any cause. The DSS is defined as the amount of time a patient survived the SGC from the start of treatment until death was confirmed or presumed to be caused by the disease itself. The cause of death was determined from the medical record review of our institution and/or follow-up information from the referring veterinarian medical records, including physical examination and clinical assessment by veterinarian(s), the results of diagnostic tests, and necropsy findings, if available. If the cause of death was unknown or for “quality-of-life” reasons, then these deaths were attributed to salivary gland carcinoma.

Dogs were censored from outcome data if they were alive at the time of manuscript preparation or were lost to follow-up. Kaplan–Meier analysis was used to estimate and display the distribution of PFI and MST and DSS. All statistical analyses were performed using commercial Prism software v8.4.3 (GraphPad Software, San Diego, CA, United States).

## 3. Results

### 3.1. Patient demographics

**Figure 1:**
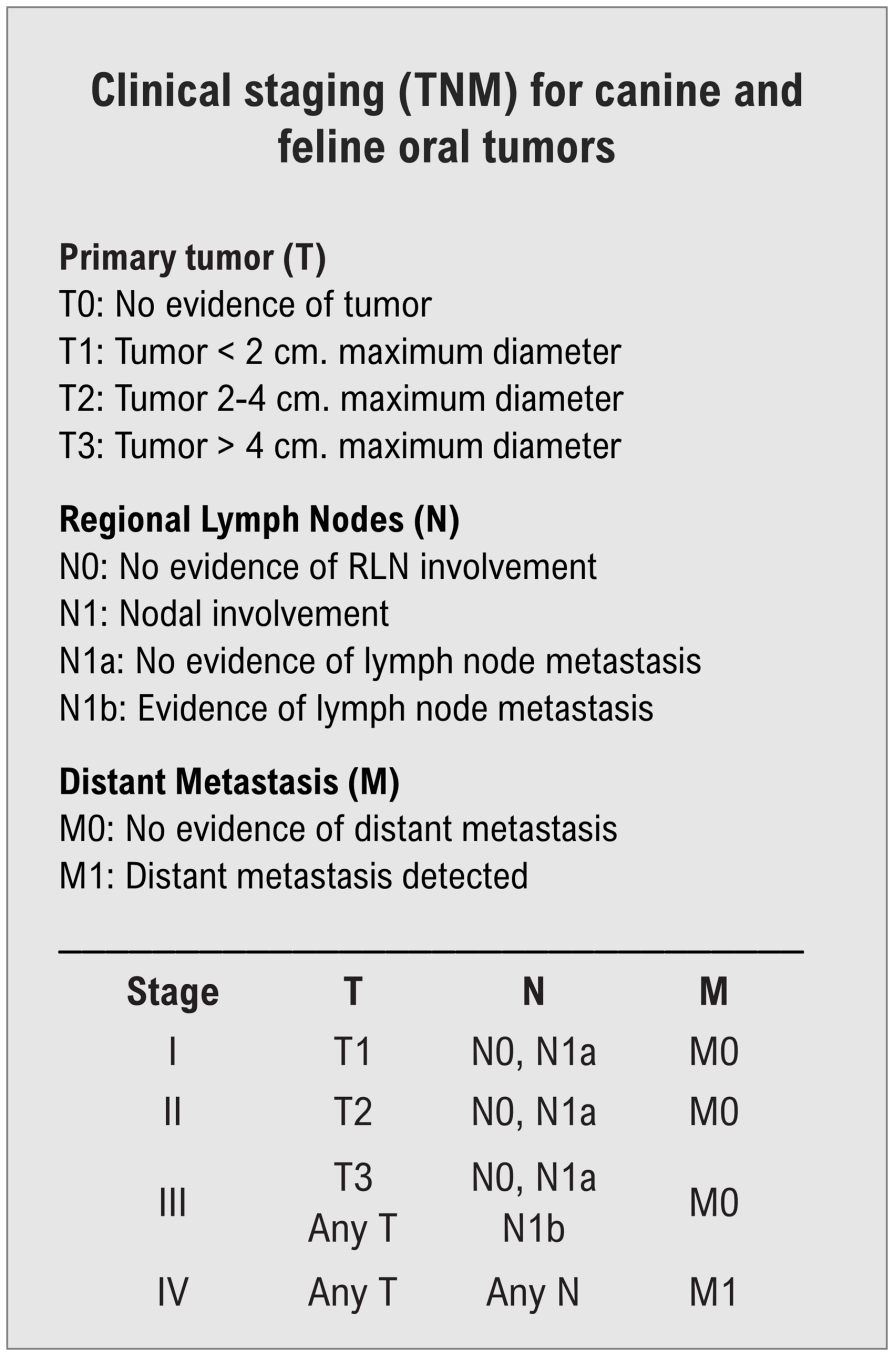
Tumor, node, metastasis (TNM) clinical staging system for oral tumors in animals, adapted from Owen, 1980 (14).

Six patients met the inclusion criteria for this study. Full patient demographics and presenting clinical signs are summarized in Table 1. Diagnosis was confirmed via cytology in 5/6 dogs (83.3%) and via incisional biopsy in 2/6 (33.3%) dogs. Concurrent comorbidities included heart disease (*n* = 4, 66%), laryngeal hemiplegia (*n* = 1, 16.6%), hypothyroidism (*n* = 1, 16.6%), Cushing’s disease (*n* = 1, 16.6%), and keratoconjunctivitis sicca (*n* = 1, 16.6%). In one of the patients (Case 5), a nasal carcinoma and intracranial meningioma were diagnosed at the same time as the salivary tumor; the nasal carcinoma was treated with 10 Gy × 3 SBRT protocol and the meningioma with 8 Gy × 3 SRT protocol concurrently with the salivary gland tumor. In another case (Case 6), a pituitary macroadenoma with associated Cushing’s disease and basal cell tumor located over the hock were diagnosed at the same time as the salivary gland carcinoma; the basal cell tumor was surgically excised at the same time of SBRT for the SGC, while medical management for Cushing’s disease associated with the pituitary macroadenoma was recommended at follow up with primary care veterinarian. None of the dogs received curative-intent surgery or chemotherapy for the salivary gland carcinoma prior to initiation of radiation therapy. At the time of SBRT, two dogs were being treated with non-steroidal anti-inflammatories, one dog with intracranial meningioma was treated with steroids (prednisolone 0.5 mg/kg/day), and the remainder of dogs were not treated with concurrent systemic anti-inflammatory medication (See Figure 1).

**Table 1:** Patient demographics and staging results.

	Case 1	Case 2	Case 3	Case 4	Case 5	Case 6
Age	10 years	11 years	12 years	13 years	13 years	12 years
Gender	MC	FS	FS	FS	FS	FS
Breed	Chihuahua	Mixed breed dog	Kelpie	Mixed breed dog	Boxer	Cocker Spaniel
Weight	6.9 kg	5.2 kg	23.8 kg	27.3 kg	19.3 kg	15.2 kg
Primary tumor location	L mandibular SG	R mandibular SG	L mandibular SG	L parotid SG	L parotid SG	L zygomatic SG
Presenting complaint	Cervical swelling	Cervical swelling	Cervical swelling	Ear base mass	Ear base mass	Retrobulbar mass
Clinical signs	Pain, dysphagia	None	Dysphagia	None	None	Exophthalmos
Cytological diagnosis	Salivary gland carcinoma	Carcinoma	Anaplastic carcinoma	Carcinoma	Carcinoma	Carcinoma
Histopathologic diagnosis	No	Carcinoma, probable salivary	No	Mucoepidermal adenocarcinoma	Salivary adenocarcinoma	No
Lymph node enlargement	Yes	Yes	Yes	No	No	Yes
Lymphadenopathy on CT	Yes	Yes	Yes	Yes	Yes	Yes
Lymph node metastasis	Yes	Yes	Suspected	Suspected	No	No
Pre-RT thoracic imaging	Radiographs	Radiographs, CT	Radiographs	Radiographs, CT	Radiographs, CT	CT
Pulmonary metastasis	No	No	No	Yes	No	No
Pre-RT abdominal imaging	No	CT	AUS	No	CT	CT, AUS
TNM Staging	T3N1bM0	T3N1bM0	T3N1aM0	T3N1aM1	T2N1aM0	T2N1aM0
Comorbidities	None	R laryngeal hemiplegia, MVD-B1	None	Ventricular ectopy, mild hypertrophy, ↓systolic function	Nasal tumor (CA), meningioma, MVD-B1, hypothyroidism	Pituitary macroadenoma, basal cell tumor (hock), Cushing’s, KCS, MVD-B1

MC, male castrated; FS, female spayed; L, left; R, right; SG, salivary gland; CT, computed tomography; AUS, abdominal ultrasound; MVD, myxomatous valve disease; CA, carcinoma; KCS, keratoconjunctivitis sicca.

### 3.2. Staging and imaging findings

**Figure 2:**
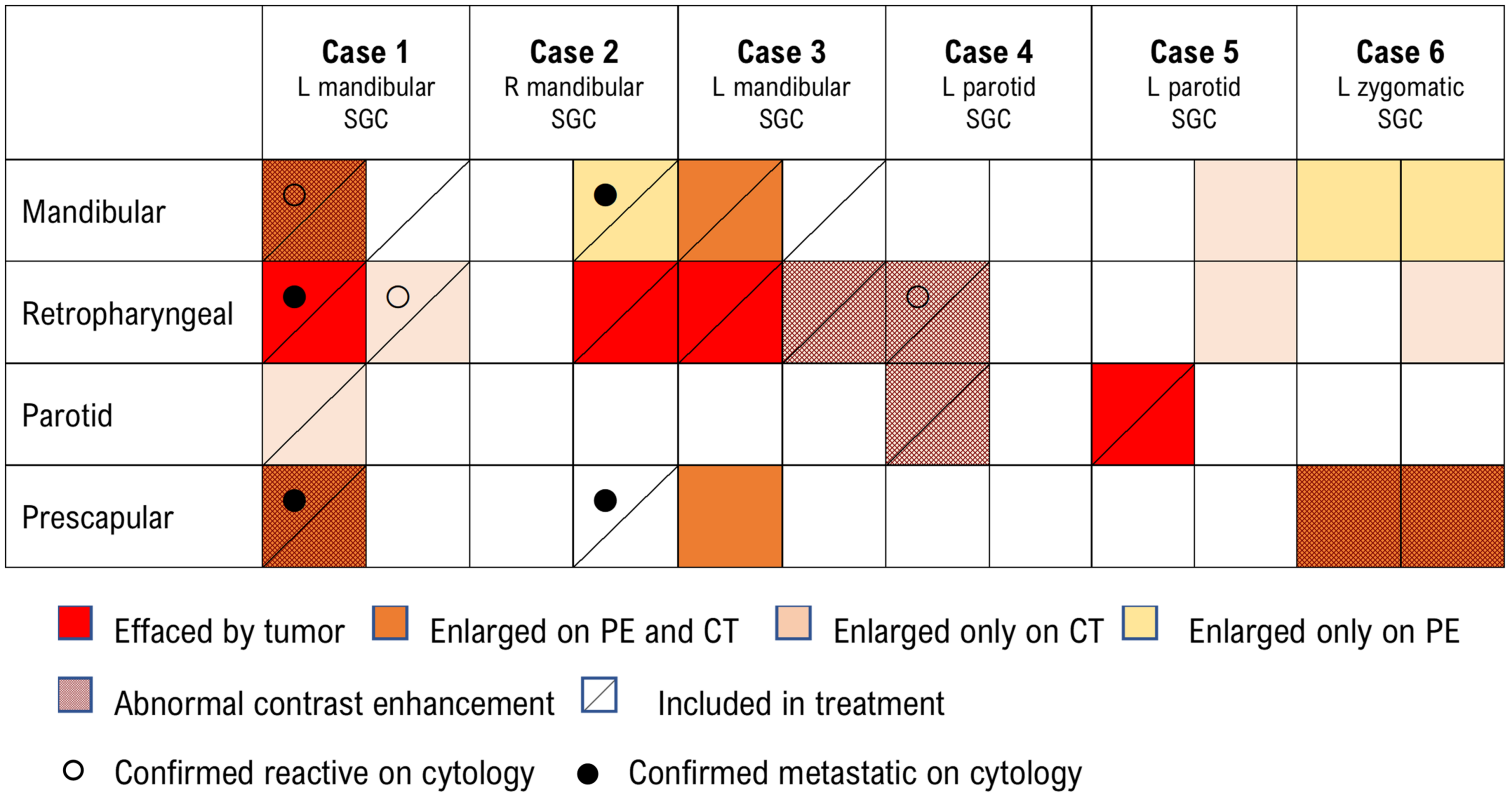
Summary of lymph node findings on physical exam, CT scan, and results of cytologic evaluation of samples obtained via fine needle aspirate. The two columns under each case indicate right-sided (right) and left-sided (left) draining locoregional lymph nodes. All lymph nodes that are included in treatment (diagonal line) and not effaced by the tumor (red) nor confirmed metastatic (●) should be considered ‘*prophylactically irradiated*’. L, left; R, right; SGC, salivary gland carcinoma; PE, physical examination; CT, computed tomography.

All dogs had a head/neck pre- and post-contrast CT scan with radiation planning. The mean longest tumor diameter on CT was 4.8 ± 1.2 cm (range 6.4–2.9 cm). Two dogs had evidence of intramuscular invasion and one dog had evidence of a tumor thrombus in the ipsilateral carotid artery. Lymph node abnormalities (e.g., enlargement, abnormal contrast enhancement pattern, effacement by tumor) were present in all patients based on advanced imaging. The lymph nodes affected included a combination of mandibular, retropharyngeal, parotid, and prescapular lymph nodes, and the extent of nodal involvement for each case is further described in Figure 2.

Two dogs with mandibular salivary gland tumor origin had confirmed locoregional metastatic disease on cytology. All dogs had thoracic staging. Evaluation of non-contrast thorax CT scan was available in 4/6 cases (66.6%); three-view radiographs were available in 5/6 cases (83.3%). One dog (Case 4) had presence of pulmonary nodules identified on CT scan prior to treatment. Abdominal imaging prior to RT was available for four patients via CT (3/4) or abdominal ultrasound (1/4), revealing no significant abnormalities. Based on TNM staging system (14), two dogs were considered stage II (33.3%), three dogs were considered stage III (50%), and one dog stage IV (16.6%) prior to initiation of treatment.

### 3.3. Radiation treatment, planning, and dosimetry profile

The first fraction of SBRT was administered in the range of 17–103 days after definitive diagnosis. Five patients were prescribed 10 Gy × 3 daily fractions that were administered on three consecutive business days, while one patient (Case 2) was prescribed 10 Gy × 3 on an every-other-day schedule, which was administered over 7 days in total. Static beams (8–11 beams) were used in three cases and VMAT (two partial or full arcs) was used in the other three cases. Lymph nodes were included in the radiation treatment plan as described in Figure 2. Regional lymph nodes were prophylactically irradiated due to clinical concerns in the absence of confirmed metastatic disease in three dogs (Case 1, Case 3, and Case 4, Figure 2).

**Figure 3:**
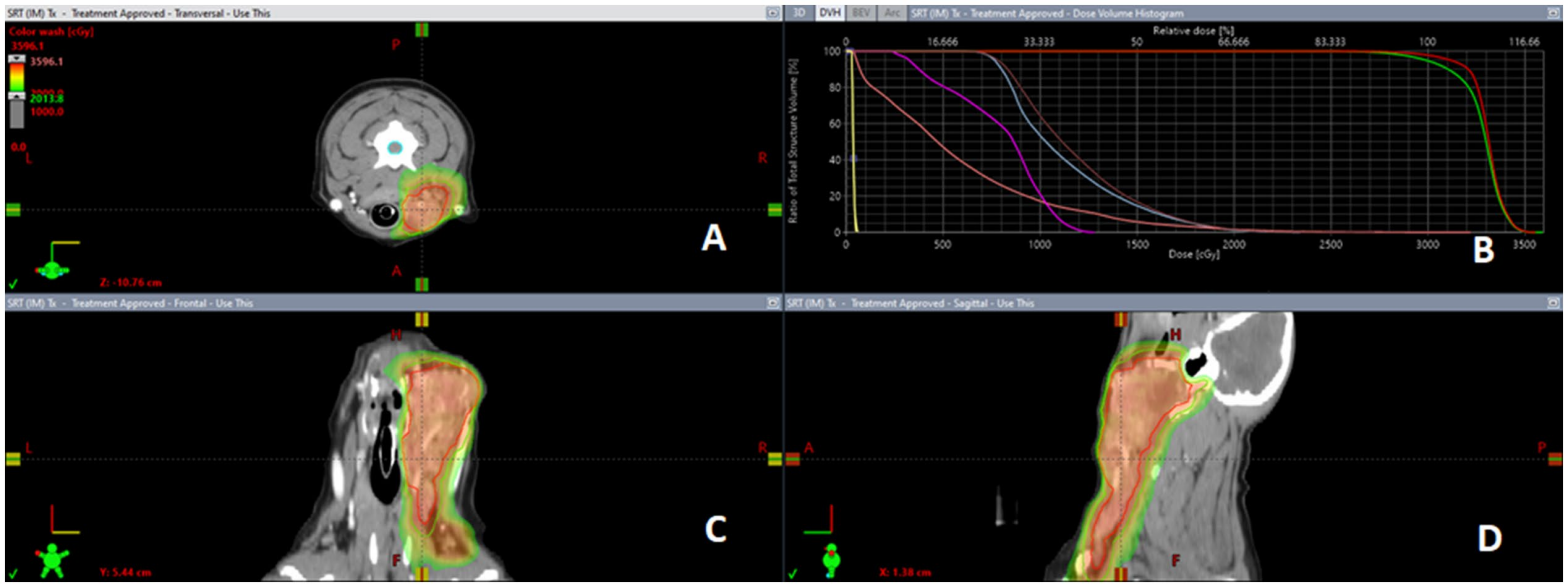
Representative salivary gland carcinoma SBRT plan dose color wash. CT guided radiation treatment plan for a case of canine SGC representing the distribution of the radiation via dose color wash in (panel **(A)**) transverse, (panel **(C)**) sagittal, and (panel **(D)**) frontal views with (panel **(B)**) corresponding dose volume histogram (DVH) for treated tumor volumes and organs at risk.

Contouring and inclusion/exclusion of metastatic or prophylactically irradiated lymph nodes in primary tumor GTV and PTV was at the discretion of the attending radiation oncologist (Figure 2). Due to the retrospective nature of the study, lymph nodes were inconsistently contoured as separated structures or included within primary tumor volume targets, at the discretion of the attending radiation oncologist (See Figure 3).

Radiation treatment dosimetry data are summarized in Table 2. The mean 99% GTV dose was 27.8 ± 1.9 Gy (range: 25.9–31 Gy), while the mean 95% PTV-less dose was 27.3 Gy ± 2.6 (range: 23.2–30.1 Gy). The intended prescription was not achieved in all cases due to considerations for the dose constraints for organs at risk (OAR). Institutional constraints used for OAR treated with a three-fraction SBRT protocol, as well as delivered dose to the OAR and reported side effects, are summarized in Table 3.

**Table 2:** Primary target volumes dosimetry data.

	Case 1	Case 2	Case 3	Case 4	Case 5	Case 6	Mean	SD	Range
Volume of GTV (cm^3^)	60.2^*^	53.6^*^	75.2^*^	65.9^*^	11.8	18.5	38.9 cm^3^	±23.9	11–75.2 cm^3^
Dose to 99% of GTV	26.2	28.9	26.3	28.6	25.9	31.0	27.8 Gy	±1.9	25.9–31 Gy
Max dose to GTV	36.8	35.6	33.9	35.2	33.6	33.5	34.7 Gy	±1.2	33.6–36.8 Gy
Min dose to GTV	20.4	25.2	17.4	23.8	23.3	29.2	23 Gy	±3.7	17.4–19.2 Gy
Mean dose to GTV	32.1	33	31.6	31.7	31.7	32.2	31.9 Gy	±0.6	31.7–33 Gy
Median dose to GTV	32.5	33.1	31.8	31.7	31.2	32.1	32.1 Gy	±0.5	31.2–33.1 Gy
Volume of PTV-less (cm^3^)	76.9	79.8	168.6	86.8	22.9	27.4	61.9 cm^3^	±48	22.9–168 cm^3^
Dose to 95% of PTV-less	25.4	29.8	23.2	29.1	26.3	30.1	27.3 Gy	±2.6	23.2–30.1 Gy
Max dose to PTV-less	36.8	35.9	33.9	35.2	33.3	33.5	34.8 Gy	±1.3	33.3–36.8 Gy
Min dose to PTV-less	14.9	23.3	12.4	22.2	23.3	23.9	19.5 Gy	±4.9	12.4–23.9 Gy
Mean dose to PTV-less	31.1	32.7	30	31.4	30.7	31.8	31.3 Gy	±0.8	30–32.7 Gy
Median dose to PTV-less	32.1	32.9	31.2	31.5	31.4	32	31.9 Gy	±0.6	31.2–32.9 Gy
HI	0.35	0.2	0.41	0.19	0.24	0.11	0.23	±0.1	0.11–0.41
CI	0.73	0.86	0.63	0.82	0.62	0.92	0.75	±0.1	0.62–0.92
GM	1.43	1.05	1.59	1.04	2.1	0.79	1.27	±0.4	1.04–2.1

GTV, gross target volume; PTV, planned target volume; HI, homogeneity index; CI, conformity index; GM, gradient measure; ^*^, GTV included LN target volumes.

**Table 3:** Normal tissue constraints and administered dose to organs at risk (OARs).

Organ at risk	SBRT constraints (3 fractions)	Volume constraints	Vol max (Gy)	Max Point dose (Gy)	Highest VRTOG score reported
Skin/mucosa	Constraint	<3 cm^3^	24 Gy		VRTOG grade 2 (acute)
	Administered	0.99 cm^3^ (0.2–1.74 cm^3^)		30 Gy (27.5–31.4 Gy)	
	Constraint			Breakthrough < 24 Gy	
	Administered			22.8 Gy (15–24.7 Gy)	
Pharynx/Trachea	Constraint	<4 cm^3^	15 Gy	30 Gy	None reported
	Administered	0.84 cm^3^ (0.1–5.5 cm^3^)		21.3 Gy (18–27.2 Gy)	
Esophagus	Constraint	<5 cm^3^	17.7 Gy	25.2 Gy	None reported
	Administered	0.17 cm^3^ (0–0.47 cm^3^)		18.5 Gy (0–23.4 Gy)	
Eyes	Constraint	Mean < 8–9 Gy	30 Gy		Unilateral blindness
OS	Administered	0.45 Gy (0.2–7.6 Gy)		0.7 Gy (0.3–30.8 Gy)	
OD	Administered	0.44 Gy (0.2–7.4 Gy)		0.6 Gy (0.3–13.3 Gy)	
Optic chiasm	Constraint			21 Gy	Unilateral blindness
	Administered			11.8 Gy (2.3–29.3 Gy)	
Brain	Constraint	<1.1 cm^3^	24 Gy	N/A	None reported
	Administered	0.1 cm^3^ (0–1.1 cm^3^)		25.6 Gy (17.3–32-2 Gy)	
Spinal cord	Constraint	<0.35 cm^3^	18 Gy	21.9 Gy	None reported
	Administered	0 (0– < 0.1 cm^3^)		15.4 Gy (0.1–18 Gy)	
	Constraint	<1.2 cm^3^	12.3 Gy		None reported
	Administered	0.75 cm^3^ (0–2.17 cm^3^)			

Volume: median (range) cm^3^; Max dose: median (range) Gy. OS, left eye; OD, right eye.

### 3.4. Treatment response, toxicity, and clinical follow up

**Figure 4:**
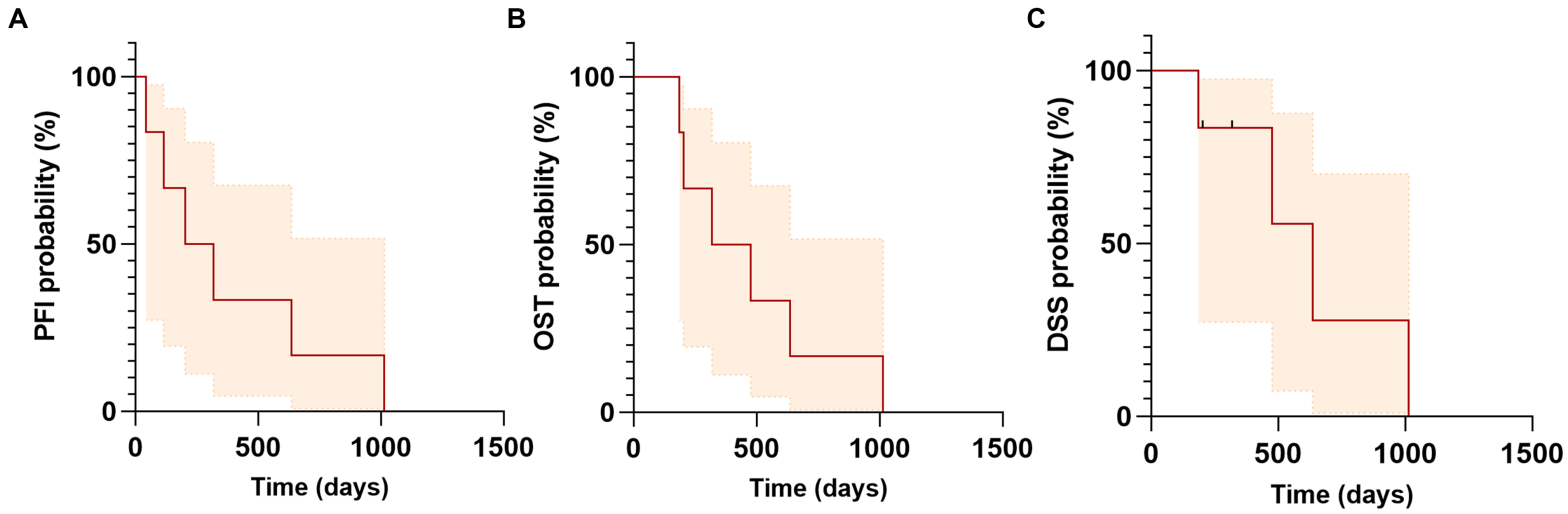
Kaplan–Meier curves of progression free interval (PFI), overall survival time (OST) and disease specific survival (DSS) for dogs with salivary gland carcinoma treated with SBRT. **(A)** Median PFI was 260 days (range: 43–1,014). **(B)** Median OST or MST was 397 days (range: 185–1,014 days). **(C)** Median DSS was 636 days (range: 185–1,014 days). Tick marks indicate time of censoring; shaded regions indicate 95% confidence intervals.

Follow-up data with at least one physical examination by a veterinarian were available for all dogs. The median follow-up period post-radiation was 397 days (range: 200–1,044 days). Amongst all patients, only one dog (Case 2) developed treatment-associated complications, which included colitis and regurgitation during the SBRT treatments. These were attributed to stress and/or anesthesia and managed with oral medications. Only two dogs (Case 1 and Case 4) were evaluated at the recommended two-week follow-up examination after completion of RT. At the two-week recheck, neither Case 1 nor Case 4 had evidence of acute toxicity. Case 4 was noted to have VRTOG grade 2 mucositis affecting the caudal oral cavity at the four-week recheck and was prescribed oral NSAID and analgesic medications (carprofen 2 mg/kg BID, gabapentin 7.7 mg/kg TID). The patient with the left-sided zygomatic SGC (Case 6) was reported to have developed left-sided blindness within 1 month of completion of RT. This dog was affected by a pituitary macroadenoma and received a mean dose of 15.6 Gy (min 8.4 – max 29.3 Gy) to the optic chiasm and mean dose of 7.6 Gy (min 0.8 – max 30.8 Gy) to the left eye. In this case, the radiation dose to the tumor was not jeopardized to spare the optic chiasm and both OAR exceeded normal tissue constraints (See Figure 4).

All dogs (100%) experienced clinical benefits; however, caliper measurements or imaging-based tumor measurements were not available, thus RECIST criteria (22) could not be applied. Based on medical record review and client interview in five of the six cases (83.3%), the median time-to-improvement was 34 days (range 28–214 days). None of the dogs were reported to have recurrent clinical signs or progression of local disease after completion of RT. None of the dogs had follow up advanced imaging. No late toxicities were reported.

Two dogs (Case 1 and Case 4) in this study were treated with adjuvant chemotherapy for their SGC following SBRT treatment. One of the two dogs had confirmed lymph node metastasis in the ipsilateral retropharyngeal and prescapular lymph nodes (Case 1) and the second dog was diagnosed with pulmonary nodules consistent with metastasis prior to start of SBRT (Case 4). Both had progressive metastatic disease at distant site (lung) confirmed via imaging at days 43 (Case 1) and 114 (Case 2) after treatment with one and four doses of carboplatin (240–300 mg/m^2^), respectively. Four dogs in this study had follow-up thoracic imaging with thoracic radiographs (range: 32–313 days post-RT). The median progression free interval (PFI) was 260 days (range: 43–1,014), with three dogs progressing with distant metastasis at 43, 114, and 636 days; the fourth patient did not have thoracic imaging and was euthanized for poor quality of life. None of the patients had local progression. The overall median survival time (MST) was 397 days (range: 185–1,014 days). The median disease specific survival (DSS) was 636 days (range: 185–1,014 days). Two dogs were censored from the DSS time analysis: Case 5, which was concurrently treated for nasal and brain tumors, was euthanized 279 days after treatment due to uncontrolled seizures and poor quality of life, while Case 6, which was originally diagnosed with pituitary macroadenoma and Cushing’s disease, experienced seizures, gastro-intestinal bleed, and heart failure and died of cardiopulmonary arrest 318 days after completion of SBRT with no evidence of disease associated with the treated salivary gland carcinoma based on physical exam and staging with thoracic radiographs and abdominal ultrasound; however, necropsy was not performed. One dog (Case 4) underwent necropsy at 476 days post-SBRT. The previously irradiated primary SGC site was described as expanded by multifocal-to-coalescing spherical nodules approximately 0.2–0.4 cm in diameter. Histologically, it was confirmed as high grade mucoepidermal adenocarcinoma, with rafts of neoplastic cells present within vessels. Metastatic SGC lesions were disseminated throughout all lung lobes, focally in the jejunum, and within the cerebrum, which were identified as the cause for the neurologic signs leading to humane euthanasia. Outcomes are summarized in Table 4.

**Table 4:** Outcomes for patients diagnosed with salivary gland carcinoma treated with SBRT.

	Case 1	Case 2	Case 3	Case 4	Case 5	Case 6
Primary tumor location	L mandibular SG	R mandibular SG	L mandibular SG	L parotid SG	L parotid SG	L zygomatic SG
Lymph node metastasis	Yes	Yes	Suspected	Suspected	No	No
Pulmonary metastasis	No	No	No	Yes	No	No
TNM Staging pre-RT	T3N1bM0	T3N1bM0	T3N1aM0	T3N1aM1	T2N0M0	T2N0M0
Prophylactic LN RT	Yes	No	Yes	Yes	No	No
RT side effects	No	No	No	Mucositis	No	Blindness, alopecia
Follow up chemotherapy	Yes	No	No	Yes	No	No
If yes, which one(s)?	CARBO, DOX			CARBO, CTX, TOC, MTX, DOC		
PFI (days)	43	636	1,014	114	202	318
OST (days)	185	636	1,014	476	202	318
DDS (days)	185	636	1,014	476	–	–
Reason for death	(E) Poor QOL	(E) Poor QOL, cough	(E) Poor QOL	(E) Seizures, vestibular events	(E) Seizures	(D) Heart failure, seizures
Necropsy	No	No	No	Yes	No	No
Comments	PD pulmonary	PD pulmonary		Confirmed lung, brain, jejunal metastasis	Nasal CA and meningioma concurrent RT	NED on CXR/AUS one day prior to death

SG, salivary gland; QOL, quality of life; PD, progressive disease; E, euthanized; D, died; PFI, progression free interval; OST, overall survival time; DDS, disease specific survival; CARBO, carboplatin; DOX, doxorubicin; CTX, cyclophosphamide; TOC, toceranib; MTX, mitoxantrone; DOC, docetaxel; CA, carcinoma; CXR, chest x-rays; AUS, abdominal ultrasound.

## 4. Discussion

Salivary gland neoplasia is considered a rare disease in domestic animals and there is sparse information in the veterinary literature regarding the clinical characteristics, treatment management, and outcome of dogs with salivary gland tumors. This is the first case series reporting on the outcome of macroscopic canine SGC treated with external beam RT. Specifically, we evaluated the feasibility and safety profile following SBRT for treatment of canine SGC in the macroscopic disease setting. In our study, SBRT was used for the treatment of locally advanced and invasive primary salivary gland carcinomas as well as treatment of confirmed or suspected locoregional metastatic disease. The results indicate that SBRT achieved a durable locoregional response in the treated dogs. In the patient where necropsy and histopathological evaluation was available, residual disease was noted in the primary SGC site, with multiple mitotic figures counted (15 mitosis in 10 representative 400× fields). While it is not possible to determine in this case if this finding represents neoplastic tissue that has fully maintained capabilities of proliferation, invasion, and metastasis following RT, this raises the question of whether a good clinical response in terms of size reduction of the primary mass is sufficient to obtain control of the disease. Patterns of failure included progression of metastatic disease at distant sites (lungs) documented in three dogs (50%) and presumed in one dog (16.6%), with the remainder of cases (33.3%) dying of unrelated causes. Although the original intention was to use RECIST criteria to assess tumor response, this was not possible due to tumor location and/or medical record limitations. Despite the lack of objective data describing tumor response, none of the patients were reported to fail at the primary tumor site or at the level of locoregional lymph nodes in the follow-up period. The finding of a mass by the owner was the most common presenting complaint in our population, as previously reported. Exophthalmos has been reported as the primary complaint for zygomatic salivary tumor location (24) and is consistent with the findings in the single case of zygomatic salivary tumor in this study. The mass-effect and associated clinical signs described at presentation were not described in the post-SBRT medical records or follow up client interview for any of the patients.

In this study, 4/6 of patients (66.6%) received prophylactic regional lymph node irradiation at the discretion of the attending radiation oncologist. In veterinary medicine, prophylactic nodal irradiation has been described for different tumor types, such as high-grade mast cell tumors in dogs (25, 26), feline nasal lymphoma (27), and oral malignant melanoma (28). However, there is not a standard consensus for elective nodal irradiation in veterinary radiation oncology. In light of the complexity of lymphatic drainage and metastatic spread patterns described in head and neck malignancies (29), the use of sentinel lymph node mapping techniques has the potential to provide a useful and non-invasive tool to identify target lymph nodes (30, 31). For human patients, the lymphatic spread of head and neck cancer (HNC) is well documented and relatively predictable, allowing for the proposal of international consensus guidelines and specific recommendations to guide unilateral vs. bilateral nodal treatment based on specific tumor type and location, nodal status, and SPECT/CT findings (32–34). This information is unfortunately unavailable in veterinary oncology and further investigation is warranted to determine the best treatment approach. Additionally, new data from mice, human, and canine species (35) has shown how the role of draining lymph nodes is essential for a response to SBRT and immunotherapy and that elective nodal irradiation is associated with a systemic decrease in circulating T cells and likely systemic immune response.

Given the lack of sensitivity and reliability of nodal staging via non-invasive methods such as physical exam, advanced imaging, and cytology in veterinary medicine (36–39), bilateral nodal irradiation of the neck may be justified in order to include undetected metastatic disease, which can have prognostic value in certain tumor types affecting the head and neck, such as canine oral malignant melanoma (39). On the other hand, considerations need to be made regarding increased normal tissue toxicity, especially for SBRT and increased potential for late side effects, as well as regarding irradiation of normal lymphatic tissue and modification of the first-line tumor immune response represented by the locoregional lymph nodes.

In our study, all dogs underwent CT scans of the head and neck for radiation planning; however, complete staging with locoregional lymph node aspirates was lacking and may have affected nodal stage. Physical exam or CT-based lymph node abnormalities (including enlargement, effacement, and abnormal contrast enhancement pattern) did not trigger investigation in all cases, but rather the sampling was based on the clinical judgement of the attending clinician. Thoracic staging was available in all cases; however, it was performed with CT in only 4/6 cases (66.6%). This may have also affected the pre-RT staging of the patients, since radiographs may detect as low as only 9% of CT-detected pulmonary nodules (40).

Advanced-stage disease (stage III or IV) was described as a significant negative prognostic factor for dogs in one previous study of canine salivary gland carcinoma (13) and was common in our study population (3/6 cases, 50%) at the time of presentation. Two dogs with advanced stages (Case 1 and 4) underwent chemotherapy with various systemic agents and progressed distantly at days 43 and 114 after treatment, respectively. Case 1 also received nodal irradiation. Case 2 also had advanced-stage disease and did not receive systemic chemotherapy, but nodal irradiation was performed. This case was reported to have developed progressive pulmonary metastatic disease over 21 months after treatment. These examples highlight the lack of information regarding prognostic factors for the development of metastatic disease, even in presence of adequate locoregional tumor control.

The treatment-associated toxicity was low. One dog (16.6%) experienced acute VRTOG grade 2 oral toxicity. Another dog (16.6%) experienced unilateral blindness, which could be expected based on the primary tumor location (zygomatic) and the ocular and optic chiasm dosimetry profile exceeding constraints; however, it may also represent a consequence of the patient’s untreated pituitary macroadenoma. Unfortunately, further details regarding ophthalmic or neurologic exam findings and the duration of clinical signs in the latter dog were not available for review. The incidence of acute toxicity data may be underestimated due to the lack of consistent 2 week and 4 week post-RT physical examinations. The incidence of late toxicity may also be underestimated due to the retrospective nature of the study and limitations of physical exam summaries in records.

### 4.1. Limitations

This paper has several limitations, mostly associated with the retrospective nature of the study. The case population was very limited; however, this can be expected given the rare nature of this disease. Definitive histopathological diagnosis confirming salivary origin was not available for all cases. There was lack of consistent lymph node and thoracic staging pre-RT, which may have affected the clinical stage of the disease and inclusion of affected lymph nodes in the radiation treatment plan. There was lack of consistent contouring for target volumes and subjective use of prophylactic nodal irradiation; however, the impact of this approach on the effectiveness of the RT treatment and outcome for the patient is unknown. There was lack of consistent follow up post-RT, which may have affected the detection of acute and late side effects. There was no follow-up advanced imaging for local and distant restaging and a lack of objective primary tumor measurements to assess response to treatment. Necropsy evaluation was lacking for most patients and, finally, the presence of multiple comorbidities and other tumor types may have affected outcome for the dogs in our population.

## 5. Conclusion

This study suggests that SBRT may provide durable local control for invasive and locally advanced SGC in dogs. The incidence of reported acute and late toxicity was low, with evidence that specific tumor location can affect the treatment-associated toxicity. Further investigation in a larger cohort of patients is warranted to identify optimal regional and systemic treatment to address metastatic disease to further improve outcomes. Additionally, this study highlights the variable approach to prophylactic nodal irradiation even in a small cohort of patients in our single institution. The role of indirect lymphography could be considered for a more standardized approach to draining lymph node mapping, sampling, and treatment inclusion. Additional future studies should be considered to investigate the role of elective nodal RT and influence on prognosis for head and neck cancer in veterinary medicine.

## Data availability statement

The data analyzed in this study is subject to the following licenses/restrictions: the data that support the findings of this study are available from the corresponding author upon reasonable request. Requests to access these datasets should be directed to Keara.Boss@colostate.edu.

## Ethics statement

Ethical review and approval was not required for the animal study because Retrospective nature of the study for client owner dogs that consent anesthesia and RT treatment. Written informed consent for participation was not obtained from the owners because Retrospective nature of the study for client owner dogs that consent anesthesia and RT treatment.

## Author contributions

PG and M-KB contributed to the conception and design of the study. PG, SL, TM, and M-KB contributed to data collection. PG organized the database, performed statistical analysis, and wrote the first draft of the manuscript. All authors contributed to the article and approved the submitted version.

## Funding

M-KB is supported by K01 OD03109.

## Conflict of interest

The authors declare that the research was conducted in the absence of any commercial or financial relationships that could be construed as a potential conflict of interest.

## Publisher’s note

All claims expressed in this article are solely those of the authors and do not necessarily represent those of their affiliated organizations, or those of the publisher, the editors and the reviewers. Any product that may be evaluated in this article, or claim that may be made by its manufacturer, is not guaranteed or endorsed by the publisher.
